# 44. Osteogenesis imperfecta: genetics and treatment

**DOI:** 10.1093/rap/rky034.007

**Published:** 2018-09-20

**Authors:** Jonathan Pinnell, Kaushik Chaudhuri

**Affiliations:** Rheumatology, University Hospitals Coventry and Warwickshire NHS Trust, Coventry, UNITED KINGDOM

**Introduction:** Osteogenesis imperfecta is a rare genetic disorder that affects 1 in 15,000-20,000 births. It is predominantly a collagen related disorder that leads to the development of abnormal weak bones. A spectrum of clinical presentations exist that can be classified according to clinical phenotype and severity using the 1979 Sillence criteria or the 2010 Nosology and Classification of Genetic Skeletal Disorders criteria. Typical features include multiple low impact fractures, bowing of the long bones, and growth deficiency. Patients may also suffer from dentinogenesis imperfecta, hearing loss, and deformities of the chest and spine. Non-skeletal features include blue sclera and heart valve insufficiencies. Severe abnormalities can cause death perinatally.

**Case description:** TA is a Caucasian female born in 1961. She first fractured a bone, her left tibia, when she was ten months old. She was diagnosed with osteogenesis imperfecta when she was aged 1-2 years old. By nine years of age she had suffered seven further fractures to her tibias, hands, and occiput. She was thinner and shorter than other children her age and had a triangular face, severe bruising, and blue sclera. TA has eight siblings of whom three, her younger brothers and sister, also received a diagnosis of osteogenesis imperfecta. Neither of her parents exhibited any features of the condition. Investigations by social services determined that the fractures were accidental. TA was sheltered from physical activity and studied at a school for 'fragile’ children. TA began to lose her hearing when she was aged 16 and underwent a left stapedectomy due to a fixed stapes footplate. During her adult life, TA has had fewer fractures but she suffers from her old injuries. Her right scaphoid bone was surgically fused when she was 28 years old due to persistent pain from an old fracture. Previous fractures also led to a leg length discrepancy that causes back and knee pain. TA did not receive any bisphosphonate treatment until her late forties. She often forgot to take her medication and eventually stopped taking it altogether. When she was 50 a DEXA scan confirmed vertebral osteoporosis which encouraged her to take alendronic acid. However, she struggled with side effects so her treatment was changed to intravenous ibandronate. She received five years of bisphosphonates and then they were stopped because her bone mineral density had improved on DEXA scan. Now she is 57 years old and has not needed to restart a bisphosphonate as she continues to maintain her bone mineral density on DEXA scan. More importantly, she has not had a fracture for the past five years. TA has one daughter of her own who does not have osteogenesis imperfecta.

**Discussion:** TA has fairly mild osteogenesis imperfecta. However, the disease expression is unusual as the mutation appears to be new in TA’s generation and it affects four of the nine siblings. 85% of osteogenesis imperfecta cases result from COL1A1 and COL1A2 autosomal dominant mutations. These mutations typically cause mild disease but should affect every generation of a family. Interestingly TA’s parents were both unaffected. Autosomal recessive mutations that can cause osteogenesis imperfecta were discovered in 2006. Whilst clinical features can skip generations, these mutations generally cause severe disease and lack the non-skeletal features. Additionally, they would not be expected to affect such a large proportion of one generation. A truly new mutation would only affect TA, not her siblings, and as a female, she is unlikely to be affected by an X-linked mutation. Parental mosaicism, in which a proportion of one of her parents’ cells had an autosomal dominant mutation, is the most likely explanation of the inheritance pattern seen. Patients with mosaicism may be asymptomatic even if 60-80% of their bone cells are abnormal. However, they can pass the condition to their children if their germ cells contain the mutation. The management of osteogenesis imperfecta should aim to improve key outcomes such as fracture rates, physical function, pain, and quality of life. Strengthening exercises and prompt treatment of fractures remain the most effective treatments. Bisphosphonates, and other antiresorptive drugs, can improve bone mineral density but this has not been shown to improve outcomes. Teriparitide, an anabolic agent, does not reduce fractures and only improves bone mineral density in mild disease. Bone marrow transplantation can improve key outcomes. It is thought that it essentially creates genetic mosaicism allowing patients to produce enough normal collagen to prevent fractures. Cell therapy, gene targeting, and gene editing are potential future treatments.

**Key Learning Points:** Osteogenesis imperfecta is a rare genetic disorder that causes bone fragility. Osteogenesis imperfecta can be difficult to differentiate from physical abuse in children or early onset osteoporosis in adults. Osteogenesis imperfecta usually results from autosomal dominant genetic mutations but autosomal recessive, X-linked, and mosaic patterns of inheritance exist. Bisphosphonates can improve bone mineral density in osteogenesis imperfecta but have not been shown to reduce fracture rates or improve physical function, pain, or quality of life.

**Disclosure: J. Pinnell:** None. **K. Chaudhuri:** None.

